# State of gender in digital health: where we are and what needs to happen to maximize benefits for all

**DOI:** 10.4069/whn.2025.06.25

**Published:** 2025-06-30

**Authors:** Patricia N. Mechael

**Affiliations:** 1 health.enabled, Washington, DC, USA; 2 Johns Hopkins Bloomberg School of Public Health, Baltimore, MD, USA

## Introduction

Modern systems, including healthcare, have historically been built with the prototypical white male as the default, contributing to systemic inequities [1]. Many clinical trials in health research lack adequately representative samples in terms of race and gender. This lack of representation has direct health consequences, including the delayed diagnosis of cardiovascular conditions among women, due to prevention protocols being developed predominantly on male data [2].

These disparities extend to access to technology, representation in technology sectors and global health leadership, and policy development, where women and marginalized populations face significant underrepresentation. Many digital health interventions are designed to support maternal and child health. However, if women lack access to the necessary technologies, they are at risk of exclusion. The digital health ecosystem continues to reveal gender-based disparities in access to health services, digital tools, and health data leading to an increased risk of deepening health inequities [3]

Given that sex- and gender-based differences are increasingly recognized as important factors in health outcomes, the state of gender in digital health warrants close examination. While digital connectivity and access to technology such as mobile phones offers substantial societal benefits, disconnection poses serious risks. One of the most critical divides is the gender digital divide. Across low and middle-income countries, women are still 8% less likely than men to own a mobile phone. Women are now 15% less likely than men to use mobile internet [4]. There is a significant association between women’s mobile phone ownership and improved outcomes in primary health care [5], highlighting the essential role of gendered access in digital transformation.

**Figure 1.**
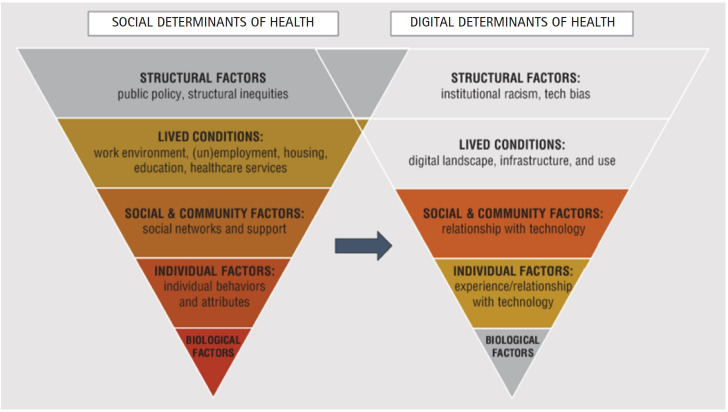
Digital determinants of health.

Although the concept of digital determinants of health has existed since the advent of technology in society, it has only recently been clearly defined [6] (Figure 1). These determinants are increasingly seen as intertwined with the social determinants of health.

## The state of gender in digital health

Gender and gender dynamics in digital health has implications across multiple domains from representation to meaningful participation in all aspects and between all levels of policy, health service delivery, demand and uptake of health services and health information among the general public, technology development and implementation, and representation within data systems and data sets [7]. The Global Digital Health Monitor (GDHM) was developed to assess the state of digital health and is currently in the process of being transitioned to the World Health Organization (WHO). In its latest iteration, the GDHM introduced Indicator 4a: *Gender considerations accounted for in digital health strategies and digital health governance*. This represents a key advancement in tracking national commitments to gender equity within the digital health landscape. However, according to the State of Digital Health Brief 2024, only 25% of the 44 countries who participated in the survey have Phase 4 or Phase 5 (out of 5 phases) maturity, meaning they implement gender-specific measures as part of their digital health governance frameworks [8].

### Digital health governance and policies

Despite advancements, women continue to be underrepresented at every level of leadership in digital health and technology policy. At the same time, the expansion of technology introduces emerging health risks, such as misinformation, threats to child safety, and technology-facilitated gender-based violence (TFGBV) [9]. These issues disproportionately affect women and call for a cautious, equity-oriented approach in the development and governance of digital health initiatives. TFGBV is on the rise, encompassing acts such as cyber harassment, cyberstalking, doxxing (revealing private information without consent), and online abuse including revenge porn and artificial intelligence (AI)-generated deepfakes. The rise of technology-facilitated gender-based violence against women highlights the dual role of technology—not only as a tool for good health and well-being, but also as a mechanism that can be exploited to exert control and perpetrate harm.

Therefore, digital health governance and policy must be thoughtfully designed and implemented to effectively prevent and respond to known negative health effects and risks. The Gender and Women’s Empowerment in mHealth framework [10] recognizes safety and power dynamics as critical intersections within technology and health, and explicitly incorporated gender-based violence as a core component. It assesses whether women are actively engaged in technology and content development, participating in policymaking, and are represented both as providers and recipients of health services. Importantly, the framework examined not only intra-group dynamics but also inter-group interactions, highlighting systemic patterns of inclusion or exclusion. Despite being introduced over a decade ago, the framework remains highly relevant, as many of the gender-related challenges it aimed to address continue to persist in today’s digital health landscape.

### Health system

Gender influences digital health interventions used by frontline health workers, who are predominantly female, and their interactions with health system supervisors and administrators who are predominantly male. Collaborative research with Gavi, the Vaccine Alliance revealed notable gender dynamics in workforce structure and gendered digital literacy gaps alongside technology ownership and use gaps [3]. It also showed gender-related gaps in data-related activities, particularly in the collection and use of data within immunization programs. To ensure effective service delivery, reach, and coverage, digital health interventions must address gendered disparities in access to digital tools and data. There are significant gender disparities in health data collection and analysis. Immunization data and digital data collection tools in health can obscure or reinforce gender bias and inequities. Even sex-disaggregated data—an essential baseline—remains underdeveloped in many settings.

### Caregivers

Digital health interventions intended to deliver health messages, reminders, and behavior change communication to caregivers (e.g., short message service [SMS] mobile phone reminders and messages, client feedback, digital vaccination records, etc.) are also impacted by gender. In many communities, household caregiving responsibilities largely rest with women, who tend to prefer voice messages over SMS for receiving health communications. Nevertheless, digital health programs are often designed and implemented by young men, which may result in misalignment with the actual preferences of their intended users [11].

The gender digital divide is also evident in areas of personal agency and health decision-making. In a mobile messaging project for maternal and child health, women responded more positively to voice messages recorded in the voice of an elderly woman. Additionally, messaging had to be adapted for other family members, such as mothers-in-law and husbands, who often serve as gatekeepers to healthcare access in patriarchal societies [12].

## Research with a gender-intentional lens

In order to meaningfully reflect on gender considerations in research implementation, digital health research studies are incorporating a gender-intentional lens. This includes engaging both male and female co-principal investigators during the design phase, ensuring gender-balanced respondent participation, and including gender-specific research questions to systematically study the relationships between gender, gender dynamics, digital health interventions and enablers, data, and health outcomes [13]. A study in Pakistan employed a comprehensive gender-specific immunization intervention [14]. Female vaccinators were proactively recruited, and the Ministry of Gender was involved in the program’s design. Gender analysis was applied throughout both the design and implementation phases. Currently in its second phase, this study aims to evaluate the impact of gender-intentional design on health outcomes compared to non-gendered interventions.

Early studies undertaken with this approach in digital health are finding that when digital health interventions apply gender-intentional approaches to design, it leads to significantly improved health outcomes. The Mapping for Health (M4H) project in the Democratic Republic of the Congo deliberately incorporated gender-intentional strategies—such as conducting a gender audit, delivering gender-focused training to stakeholders, and deploying a gender-analysis toolkit—to ensure that women were equitably involved in geospatial data collection, analysis, and immunization microplanning. As a result, the initiative not only enhanced immunization coverage and equity but also strengthened gender-responsive capacities within the national immunization program through inclusive stakeholder engagement and gender-informed decision-making [15].

By contrast, while some improvements were seen without gender considerations, the scale of improvement and coverage achieved remains limited compared to gender-intentional approaches. Another gender-intentional study conducted in Pakistan evaluated a digital health intervention that did not utilize a gender-intentional approach. The project aimed to enroll children in an immunization alert and reminder system. However, male vaccinators were responsible for requesting contact numbers from female caregivers—a practice that proved culturally inappropriate in this setting. In such cultural contexts, women are highly reluctant to share their phone numbers with unfamiliar men, even when concerning their child’s health. The study’s key recommendation was to increase the presence of female vaccinators and strengthen community engagement to enhance program acceptability [16].

## Gender-intentional digital health

In the context of digital health, it is no longer sufficient to merely acknowledge gender as a consideration. Instead, a gender-transformative approach is required—one that actively seeks to address and alter the underlying social and structural dynamics associated with gender in order to achieve true health equity [7]. Based on this understanding, the gender mHealth framework has evolved into this more systematic and comprehensive structure. The *Gender-intentional digital health interventions & enablers: A rapid guide for analysis, planning, and monitoring* [7] goes beyond superficial inclusion and seeks to embed gender equity principles into the core of digital health intervention design, implementation, and evaluation as well as into known enablers such as governance, workforce, and infrastructure.

Applying this framework, a gender-focused digital health study is currently being finalized in Ethiopia [17]. Findings indicate that Ethiopia demonstrates strong gender leadership at the national level, including an explicit commitment to achieving gender parity (50:50) in leadership roles. The country also maintains comprehensive gender policies, including a dedicated gender digital health policy and strategy [18], and has made active efforts toward institutionalizing gender mainstreaming at the highest levels of government. However, these policy advances have yet to translate into meaningful change at the subnational or community levels. Persistent barriers remain in areas requiring women’s engagement, such as technology design, supervisory positions, and policymaking roles. The study concludes that in order to realize gender transformation in digital health, further efforts are required to address societal factors that limit women’s participation and to foster inclusive structures at all levels of implementation.

## Digital health equity

Health equity is defined as the condition in which all individuals have a fair and just opportunity to attain their full potential for health and well-being [19]. However, disparities in access to technology, digital engagement, and data participation serve as critical drivers of sustained or even deepened inequity. In digital health systems, unequal access to technological resources can deepen gender-based structural inequalities, thereby undermining efforts to achieve health equity. To address this challenge, a comprehensive framework is needed—one that incorporates gender, technology accessibility, and data inclusion into all stages of digital health design and implementation to ensure meaningful progress toward equity.

### Data equity

The scope of equity in digital health must include not only technological access but also data equity. Data equity refers to a set of principles and practices to guide anyone who works with data through every step of a data project using a lens of justice, equity, and inclusivity [20]. As the use of data science and digital health applications expands, it is critical to examine who is included—and who is excluded—in the data that informs our decisions. Notably, algorithmic bias in AI has revealed substantial and troubling disparities, particularly disadvantaging women [21,22].

This underscores the urgent need for increased representation and gender sensitivity in the data ecosystem. Ensuring that data does not systematically exclude or misrepresent certain groups—especially women—requires proactive involvement of individuals who can bring these perspectives into data collection, analysis, and application. Data equity is thus an essential pillar for advancing both health equity and technological justice.

### Promoting and measuring equity in health data and large language models

Achieving algorithmic equity has become a critical priority in the application of health data and AI technologies. An increasing number of frameworks have been developed to evaluate health equity within algorithms, alongside documented cases where insufficient fairness and equity testing of large language models has led to harm. In the health sector in particular, inadequate evaluation not only of accuracy but also of equity can result in significant physical risks to individuals [23,24].

### Equity-focused artificial intelligence and machine learning

Implementing equity-centered AI and machine learning (ML) requires the use of diverse and representative datasets in order to prevent bias and ensure inclusivity [25]. Known gaps in representativeness must be clearly documented, and where appropriate, new data streams should be introduced to enhance the overall diversity of the data ecosystem-either by geography, gender, race, and/or language.

The implementation must be accompanied by continuous auditing and evaluation of embedded biases [26]. Where possible, known biases should be explicitly documented, and diverse stakeholders should be engaged to inform algorithm development and assess outcomes with an equity perspective. The development of AI solutions should prioritize fairness metrics and transparency. Partnerships should be leveraged to design and conduct experiments that test fairness measures and systematically improve outcomes. In addition, when collecting and using health data, especially from underserved communities, ethical guidelines should be established and applied, emphasizing representativeness, informed consent, and privacy protection. Advancing equity requires intersectional approaches that ensure inclusivity in research design, leadership, and policymaking structures.

## Recommendations for action

While potential biases in health and associated research are generally disclosed during early review processes such as Institutional Review Boards, much of this accountability is lost in later stages of implementation and publication. Most studies do not report algorithmic biases or mismatches in sample representation, highlighting the need for strengthened accountability, more rigorous fairness metrics, and systematic ethical standards for the collection and use of health data. These actions are essential to ensure that AI and ML function not as tools that reinforce inequality, but as mechanisms that advance equitable health outcomes. Given that health conditions often manifest differently across sexes, a gender-sensitive approach to data science is crucial. Including more women in statistics and data science is not only beneficial but necessary for the equitable advancement of health research and practice [27].

To advance health equity in practice, several actionable recommendations are proposed. These include promoting gender analysis and structured planning and monitoring from a gender perspective. Secondly, increasing gender considerations in digital health, technology, and data is needed and can begin with representation in leadership, governance, data, technology development, and algorithm development. Another key action is supporting gender-disaggregated data as well as equity and inclusion in clinical trials and health research. Finally, as a gender-transformative approach cannot be realized without first understanding the current state, setting clear objectives, and proactively navigating through implementation processes, gender-intentional and equity measurement and research on these aspects are key to ensuring their integration in ongoing work.

Comprehensive gender policies, active programming interventions, and routine gender-focused analyses—including equitable representation, workforce inclusion, and gender impact assessments in digital health will contribute to a fairer and equitable digital health ecosystem and improved health outcomes for all.

## References

[1] Criado Perez C (2019). Invisible women: data bias in a world designed for men.

[2] Mazure CM, Jones DP (2015). Twenty years and still counting: including women as participants and studying sex and gender in biomedical research. BMC Womens Health.

[3] Gavi & health.enabled (2022 Mar). Gender and digital health information in immunisation programming: Gavi Digital Health Information Strategy technical brief [Internet]. https://www.gavi.org/news/document-library/gender-and-digital-health-information-immunisation-programming.

[5] LeFevre AE, Shah N, Bashingwa JJ, George AS, Mohan D (2020). Does women's mobile phone ownership matter for health? Evidence from 15 countries. BMJ Glob Health.

[6] Chidambaram S, Jain B, Jain U, Mwavu R, Baru R, Thomas B (2024). An introduction to digital determinants of health. PLOS Digit Health.

[7] Gavi & health.enabled (2022 Oct). Gender‑intentional digital health interventions & enablers: a rapid guide for analysis, planning, and monitoring [Internet]. https://www.gavi.org/sites/default/files/programmes-impact/support/Gender-Intentional-DHI-and-Enablers-a-rapid-guide-for-analysis-planning-and-monitoring.pdf.

[8] health.enabled (2025 Mar). Global Digital Health Monitor: State of Digital Health Brief 2024. Prepared in support of the World Health Organization and Global Initiative on Digital Health [Internet]. https://digitalhealthmonitor.org/stateofdigitalhealth24.

[9] https://www.unwomen.org/sites/default/files/2024-10/a-79-500-sg-report-ending-violence-against-women-and-girls-2024-en.pdf.

[10] Deshmukh M, Mechael P (2013 Mar). Addressing gender and women’s empowerment in mHealth for MNCH: an analytical framework [Internet]. https://www.villagereach.org/wp-content/uploads/2013/07/gender_analytical_framework_report.pdf.

[11] Mechael PN, Dodowa Health Research Center (2009 Aug). MoTeCH: mHealth ethnography report [Internet]. http://www.kiwanja.net/database/document/document_motech_mhealth_ethnography_report.pdf.

[12] Lebrun V, Dulli L, Alami SO, Sidiqi A, Sultani AS, Rastagar SH (2020). Feasibility and acceptability of an adapted mobile phone message program and changes in maternal and newborn health knowledge in four provinces of Afghanistan: single-group pre-post assessment study. JMIR Mhealth Uhealth.

[13] World Health Organization (WHO), United Nations University International Institute for Global Health (2021). Integrating gender in digital health: a framework for action [Internet]. https://iris.who.int/bitstream/handle/10665/342570/WHO-EURO-2021-2318-42073-57916-eng.pdf.

[14] UNICEF (2024). Engaging female vaccinators in Pakistan: a case study [Internet]. https://www.unicef.org/media/165116/file/en-case-study-engaging-female-vaccinators-in-Pakistan-2024.pdf.

[15] Ngo-Bebe D, Mechael P, Kwilu FN, Bukele TK, Langwana F, Lobukulu GL (2025). Assessing the use of geospatial data for immunization program implementation and associated effects on coverage and equity in the Democratic Republic of Congo. BMC Public Health.

[16] Mechael P, Gilani S, Ahmad A, LeFevre A, Mohan D, Memon A (2024). Evaluating the “Zindagi Mehfooz” electronic immunization registry and suite of digital health interventions to improve the coverage and timeliness of immunization services in Sindh, Pakistan: mixed methods study. J Med Internet Res.

[17] health.enabled, Johns Hopkins Bloomberg School of Public Health Gender Equity Unit, University of Gondar Center for Digital Health and Implementation Science (2024 Nov). Gender-intentional digital health: preliminary reflections from a gender study in Ethiopia [blog on the internet]. https://digitalhealthweek.co/2024/11/05/y-reflections-from-a-gender-study-in-ethiopia/.

[19] Richardson S, Lawrence K, Schoenthaler AM, Mann D (2022). A framework for digital health equity. NPJ Digit Med.

[20] Global Future Council on Data Equity (2024 Sep). Advancing data equity: an action‑oriented framework [Internet]. https://www3.weforum.org/docs/WEF_Advancing_Data_Equity_2024.pdf.

[21] United Nations Women (2025 Feb). How AI reinforces gender bias—and what we can do about it [Internet]. https://www.unwomen.org/en/news-stories/interview/2025/02/how-ai-reinforces-gender-bias-and-what-we-can-do-about-it.

[22] Pasipamire N, Muroyiwa A (2024). Navigating algorithm bias in AI: ensuring fairness and trust in Africa. Front Res Metr Anal.

[23] Kim JY, Hasan A, Kellogg KC, Ratliff W, Murray SG, Suresh H (2024). Development and preliminary testing of Health Equity Across the AI Lifecycle (HEAAL): A framework for healthcare delivery organizations to mitigate the risk of AI solutions worsening health inequities. PLOS Digit Health.

[24] Schaekermann M, Spitz T, Pyles M, Cole-Lewis H, Wulczyn E, Pfohl SR (2024). Health equity assessment of machine learning performance (HEAL): a framework and dermatology AI model case study. EClinicalMedicine.

[25] Ueda D, Kakinuma T, Fujita S, Kamagata K, Fushimi Y, Ito R (2024). Fairness of artificial intelligence in healthcare: review and recommendations. Jpn J Radiol.

[26] https://arxiv.org/abs/2009.10576.

[27] Chinta SV, Wang Z, Palikhe A, Zhang X, Kashif A, Smith MA (2025). AI-driven healthcare: Fairness in AI healthcare: a survey. PLOS Digit Health.

